# A new pharmacological role for donepezil: attenuation of morphine-induced tolerance and apoptosis in rat central nervous system

**DOI:** 10.1186/1423-0127-21-6

**Published:** 2014-01-23

**Authors:** Mozhdeh Sharifipour, Esmaeal Izadpanah, Bahram Nikkhoo, Samad Zare, Ali Abdolmaleki, Katayoun Hassanzadeh, Farshid Moradi, Kambiz Hassanzadeh

**Affiliations:** 1 Department of Biology, Faculty of Basic Science, Urmia University, Urmia, Iran; 2 Cellular and Molecular Research Center, Kurdistan University of Medical Sciences, Sanandaj, Iran; 3 Department of Physiology and Pharmacology, Faculty of Medicine, Kurdistan University of Medical Sciences, Sanandaj, Iran; 4 Department of Pathology, Faculty of Medicine, Kurdistan University of Medical Sciences, Sanandaj, Iran; 5 Science and Research Branch, Islamic Azad University, Hamedan, Iran; 6 Student Research Committee, Kurdistan University of Medical Sciences, Sanandaj, Iran

**Keywords:** Apoptosis, Donepezil, Morphine, Tolerance

## Abstract

**Background:**

Tolerance to the analgesic effect of opioids is a pharmacological phenomenon that occurs after their prolonged administration. It has been shown that morphine-induced tolerance is associated with apoptosis in the central nervous system and neuroprotective agents which prevented apoptosis signaling could attenuate tolerance to the analgesic effects. On the other hand donepezil, an acetylcholinesterase inhibitor, has been reported to have neuroprotective effects. Therefore in this study, the effect of systemic administration of donepezil on morphine-induced tolerance and apoptosis in the rat cerebral cortex and lumbar spinal cord was evaluated. Various groups of rats received morphine (ip) and different doses of donepezil (0, 0.5, 1, 1.5 mg/kg/day). Nociception was assessed using tail flick apparatus. Tail flick latency was recorded when the rat shook its tail. For apoptosis assay other groups of rats received the above treatment and apoptosis was evaluated by *in situ* terminal deoxynucleotidyl transferase-mediated dUTP-biotin nick end-labeling (TUNEL) method.

**Results:**

The results showed that administration of donepezil (0.5, 1, 1.5 mg/kg, ip) delayed the morphine tolerance for 9, 12 and 17 days, respectively. Furthermore pretreatment injection of donepezil attenuated the number of apoptotic cells in the cerebral cortex and lumbar spinal cord compared to the control group.

**Conclusion:**

In conclusion, we found that systemic administration of donepezil attenuated morphine-induced tolerance and apoptosis in the rat cerebral cortex and lumbar spinal cord.

## Background

Prolonged exposure to opioids such as morphine results in the development of analgesic tolerance, and significantly limits the clinical usage of opioids. The neurobiological mechanisms underlying the development of opioid tolerance are multifaceted and are not completely understood. During the past decades, many studies have focused to clarify the mechanisms involved in morphine tolerance. In addition, it has been shown that apoptotic cell death would be induced in association with the development of morphine tolerance [1,2]. Apoptosis, or program med cell death, is an active process of normal cell death during development and also occurs as a consequence of the cytotoxic effect of various neurotoxins [3]. In vitro studies indicated that exposure to opioid receptor agonists increases their vulnerability to death by apoptotic mechanisms [4,5]. Other studies have demonstrated that chronic morphine administration in rats is associated with remarkable significant changes in the key proteins involved in the apoptosis signaling which collectively leads to induction of apoptosis [2,6-10].

There are several lines of evidence indicating the roles of excitatory amino acid receptors in the development of tolerance to the antinociceptive action of morphine. Numerous studies have demonstrated that N-methyl-D- aspartate receptor (NMDAR) antagonists and blockers have the ability to prevent the development of opioid-induced tolerance and dependence [11-15]. Other studies have shown that activation of NMDARs can lead to neurotoxicity under many situations [16,17]. For instance, peripheral nerve injury has been shown to activate spinal cord NMDARs, which results in neuronal cell death by means of apoptosis [18,19]. On the other hand, it has been reported that up-regulation of pro-apoptotic factors was inhibited when morphine was co-administered with the noncompetitive NMDAR antagonist, MK-801 [20]. Taking together the above studies demonstrated that there is a significant relationship between opioid-induced tolerance and apoptosis.

More recently donepezil has been found to have neuroprotective effects. Donepezil is a specific, noncompetitive and reversible inhibitor of acetylcholine–esterase (AChE). AChE inhibitors are currently used to treat Alzheimer’s disease (AD). Previous studies demonstrated that AChE inhibitors, such as donepezil and galantamine, exert a protective effect via the nicotinic acetylcholine receptor (nAChR)-mediated cascade [21,22]. In addition, it has been reported that AChE inhibitors inhibit the progress of brain atrophy in AD [23], indicating the attenuation of neuronal death in the brain of the patients.

Findings of the previous studies showed that AChE receptor inhibitors provide neuroprotection against glutamate-induced excitotoxicity by stimulating the phosphatidylinositol-3 kinase (PI3K) signaling pathway [21,24]. In the present study, we investigated the effect of donepezil on morphine-induced apoptosis in the rat cerebral cortex and lumbar spinal cord during the development of tolerance to the analgesic effects of morphine.

## Methods

### Animals

Male Wistar rats (n = 168) weighing 250–300 g were used. The animals were housed in a temperature-controlled environment (24 ± 0.5°C) and kept on a 12 h light/dark cycle (light on 08:00 am) with free access to food and water. All experiments were in accordance with the Guide for the Care and Use of Laboratory Animals (National Institutes of Health Publication No. 85–23, revised 1985) and were approved by the research and ethics committee of Kurdistan University of Medical Sciences.

### Experimental groups

Table 1. Shows the experimental groups.

**Table 1:** The experimental groups for behavioral & immunohistological evaluations

**Study sections**	**Treatment groups (n = 8 per group)**	
**Behavioral evaluation**	Saline (1 ml/kg, ip)	
	Morphine (10 mg/kg, ip) + Donepezil (0, 0.5, 1, 1.5 mg/kg, ip)	
	The most effective dose of Donepezil (1.5 mg/kg, ip) for 14 days	
	**Groups for dose–response curves:**	Saline (1 ml/kg, ip)
	Animals received opposite treatments for 14 days, on 15th day, in separate groups logarithmic doses of morphine (1 or 100 mg/kg, ip) were administered to generate analgesic dose–response curves	Morphine (10 mg/kg, ip) + donepezil (0, 1.5 mg/kg, ip)
		Morphine (10 mg/kg, ip) + MK801(0.1 mg/kg, ip)
**Immunohistological evaluation**	Saline (1 ml/kg, ip) for 14 days	
	Morphine (10 mg/kg, ip) + Donepezil (0, 0.5, 1, 1.5 mg/kg, ip) for 14 days	
	The most effective dose of Donepezil (1.5 mg/kg, ip) for 14 days	
	Morphine (10 mg/kg, ip) + MK-801(0.1 mg/kg) for 14 days	

### Drugs

Morphine sulfate (Darupakhsh, Iran) (1, 10, 100 mg/kg, ip) was dissolved in normal saline and injected using 1-ml insulin syringes. Donepezil (Sigma- Aldrich, Inc.) (0.5, 1, and 1.5 mg/kg) was dissolved in sterile 0.9% normal saline. Dizocilpine (MK801) (Sigma-Aldrich) (0.1 mg/kg) was dissolved in in sterile 0.9% normal saline. Solutions were prepared freshly on the days of the experiment.

### Induction of tolerance to the analgesic effect of morphine

To induce tolerance to the analgesic effect of morphine, rats (*n =* 8 per group) were injected with morphine (10 mg/kg, ip) once a day (at morning: 10 am) until tolerance induction. This dose has been found to cause profound analgesia with no side effects in normal rats and was also according to the our previous study [20]. For evaluation of donepezil and MK801’s effect on morphine-induced tolerance, morphine was administered 30 minutes after the intraperitoneal (ip) injection of donepezil, MK801 or their vehicle every day.

### Assessment of nociception

Nociception was assessed using a radiant heat tail flick apparatus (IITC Life Science, Woodland Hills, CA 91367, USA). The rat tail was marked with a pen about 5 cm from the tip and the light beam was focused on this marked site. A built-in Timer is automatically stopped when the animals’ tail flicks out of the beam of light, test result will be displayed on readout for viewing. The latency was recorded when the rat move its tail. Baseline tail flick latency for each rat was determined and designed as the baseline latency. The baseline latency was the average for three measurements and the intensity of the light was adjusted so that baseline latencies were 2–3 seconds. A cut-off time (10 sec) was imposed to prevent tissue damage. Tail flick response latencies (s) were expressed as the percentage of maximal possible effect (%MPE) using the equation below:

$$\begin{array}{l} \%\mathrm{MPE}=\left[\text{Post}‒\text{drug}\;\text{latency}\;\left(s\right)‒\text{Baseline}\;\text{latency}\;\left(s\right)\right]\\ \;/\left[\text{Cut}‒\text{off}\;\text{value}\;\left(s\right)‒\text{Baseline}\;\text{latency}\;\left(s\right)\right]\times 100 \end{array}$$

Baseline latency was determined once per day (average of three measurement) for each rat, before daily injection of morphine (10 mg/kg). After baseline determination, the drugs or their vehicle were administered, 30 min later the morphine was injected and finally the post-drug latency was measured after 30 min. The %MPE was then calculated for that day. Every day the baseline and latency time were registered and %MPE was calculated every day. The experiments continued until there was no significant difference in %MPE between the vehicle- or drug-treated groups (tolerant animals) and the saline group [25].

### Evaluation of tolerance induction

To evaluate the induction of tolerance, groups of rats received saline or morphine + saline or morphine + donepezil (the most effective dose) once a day for 14 days, and then logarithmic doses of morphine administrated to generate analgesic dose–response curves (according to the experimental groups table). In analgesic dose–response curves, morphine antinociceptive 50% effective dose (ED50) values for each of the drug groups were derived using linear regression of%MPE of the logaritmic doses of morphine. Differences in the ED50 estimations were determined using the confidence interval method at P <0.05 [26].

### Tissue preparation

For the in situ terminal deoxynucleotidyl transferase mediated dUTP-biotin nick end-labeling (TUNEL) assay, all animals (n = 8) received the above noted treatment regimens. On the day of tolerance completion in the control group (day 14th) and 2 h after the last dose of vehicle or treatment, the animals were euthanized by injection of ketamine and midazolam and perfused transcardially first with 0.9% saline (NaCl). Then cerebral cortexes and lumbar spinal cords were immediately dissected and fixed overnight at 4°C in the fixative solution (4% paraformaldehyde in 0.1 M phosphate buffered saline (PBS), pH adjusted to 7.4). Following fixation, these tissues were cryoprotected in 10%, 20% and 30% sucrose (in PBS) overnight at 4°C. Subsequently, the tissues were transferred to Tissue-Tek OCT (Sakura FineTek, Gardena, CA) embedding compound inside the plastic molds. The blocks were stored at -70°C until cutting time.

### Detection of apoptotic cells

After fixation and OCT embedding, the samples were cut into 3 μm-thick with a Cryocut apparatus (Leica 1800, Germany). For the TUNEL assay, an *in situ* Cell Death Detection kit (Roche Applied Science, Cat # 11 684 817 910) was used. This method allows us to examine the topographic distribution of apoptotic cells within the cerebral cortex and lumbar spinal cord. The tissue sections were stained according to the manufacturer’s instructions. Briefly, these sections were rinsed in series by fixation solution (4% paraformaldehyd in PBS, pH = 7.4) for 20 min at room temperature (RT) washing buffer (PBS) for 30 min, blocking solution (3% H_2_O_2_ in methanol) for 10 min at 15 to 25°C and permeabilization solution (0.1% triton x-100 in 0.1% sodium citrate) for 2 min on ice (2 to 8°C) to increase the permeability. After being rinsed twice in PBS, the sections pretreated with proteinase K (Roche, Germany) for 30 min at 37°C. Then, these sections were exposed to the TUNEL reaction mixture, which contains terminal deoxynucleotidyl transferase and nucleotides including fluorescein isothiocyanate-labeled dUTP for 60 min at 37°C in a dark, humidified atmosphere. After that, an anti-fluorescein peroxidase (POD)-linked antibody was added, followed by incubation for 30 min at 37°C. Finally, the reaction product was visualized by 3,3 diaminobenzidine tetrahydrochloride (DAB) incubation for 15 min at RT, and the slides were then counterstained with toluidine blue. A sub-population of apoptotic cells, scattered throughout the tissue section, was intensely stained (brown) by the TUNEL treatment. The number of apoptotic cells was counted using an Olympus IX71 microscope (40× objective) over 30 fields by a person who was blind to the treatment.

### Data analysis

Behavioral data are expressed as the mean of %MPE ± sem of eight rats per group. Student’s t-test or one-way analyses of variance followed by Tukey’s test were used to analyze statistical significance in two or multiple comparisons respectively. *P* values <0.05 were considered to be significant in all analyses.

*P < 0.05, **P < 0.01, and ***P < 0.001 indicate a significant difference as compared with the saline group for that day. The histological data (cell counting) from cerebral cortexes and spinal cords sections were averaged and are expressed as mean ± sem.

## Results

### Induction of tolerance to the antinociceptive effect of morphine

**Figure 1:**
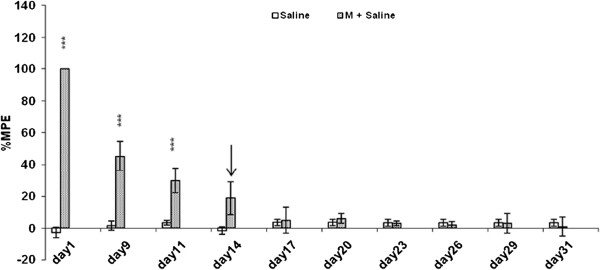
**Analgesic effect of daily administration of morphine (10 mg/kg, ip) in combination with morphine + 0 mg/kg donepezil (control group).** Developed tolerance to the analgesic effect of morphine was complete on the 14th day when there were no significant differences in percentage of maximal possible effect (%MPE) between the control groups and the saline group. Each bar represents the mean of%MPE ± sem for eight rats. M = Morphine.

As shown in Figure 1, daily administration of morphine (10 mg/kg, ip) induced tolerance to the antinociceptive effect of this drug in the control group (morphine + 0 mg/kg donepezil). The analgesic effect of morphine decreased on the 14th day, because there were no significant differences between the control groups and the saline-treated animals on the day 14, this was considered the day of morphine tolerance completion.

### Evaluation the effect of donepezil on morphine-induced tolerance to the analgesic effect

**Figure 2:**
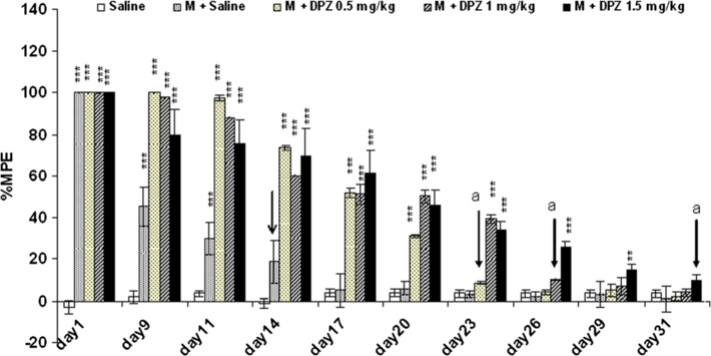
**Effect of daily systemic injections of donepezil (0, 0.5, 1, 1.5 mg/kg, ip) on tolerance to the analgesic effect of morphine.** Each bar represents the mean of percentage of maximal possible effect (%MPE) ± sem for eight rats. Student’s t-test was used to analysis the differences of %MPE between saline and treated animals every day also it was utilized for comparison of the day of tolerance establishment in control or treatment groups. *P < 0.05; **P < 0.01; ***P < 0.001 when compared with the saline group. a = P < 0.001 when compared with the control group. Arrow represents the day of morphine tolerance. M = Morphine; DPZ = Donepezil.

**Figure 3:**
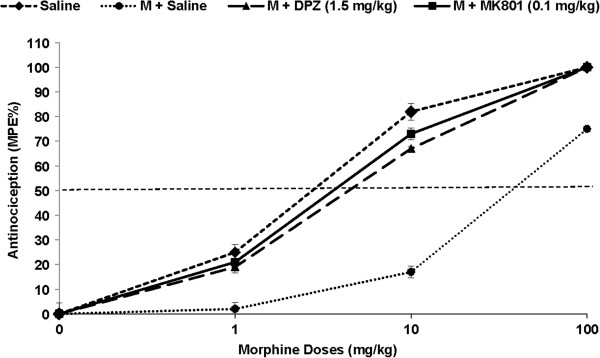
**Tail flick responses and percent of maximal possible effect (%MPE) to logarithmic doses of morphine (1, 10, 100 mg/kg, ip) administered on Day 14 after a 13 days continuous systemic drug administration.** Each point represents the mean ± sem for 8 rats. Different morphine doses administered on day 14 demonstrated a significant shift to the right in the dose–response curve and antinociceptive ED_50_ values in animals treated with morphine + 0 mg/kg donepezil compared with those receiving saline or morphine + donepezil (1.5 mg/kg, ip) or morphine + MK801 (0.1 mg/kg, ip). M = Morphine; DPZ = Donepezil.

Donepezil (0.5, 1, 1.5 mg/kg, ip) delayed the onset of morphine-induced tolerance for 9, 12 and 17 days, respectively (Figure 2). In addition, the results in Figure 3 show a significant shift to the left in the dose–response curve for animals who received morphine + donepezil compared with those receiving morphine + 0 mg/kg donepezil as control. A significant shift to the left in ED50 in the morphine + donepezil (1.5 mg/kg, ip) (32.2 mg) or morphine + MK801 (0.1 mg/kg, ip) (29.7 mg) treated groups compared with the control animals (64.5 mg) was also seen. The results also showed that the most effective dose of donepezil (1.5 mg/kg, ip) had no significant analgesic effect (data not shown).

### Effect of donepezil on morphine-induced apoptosis

**Figure 4:**
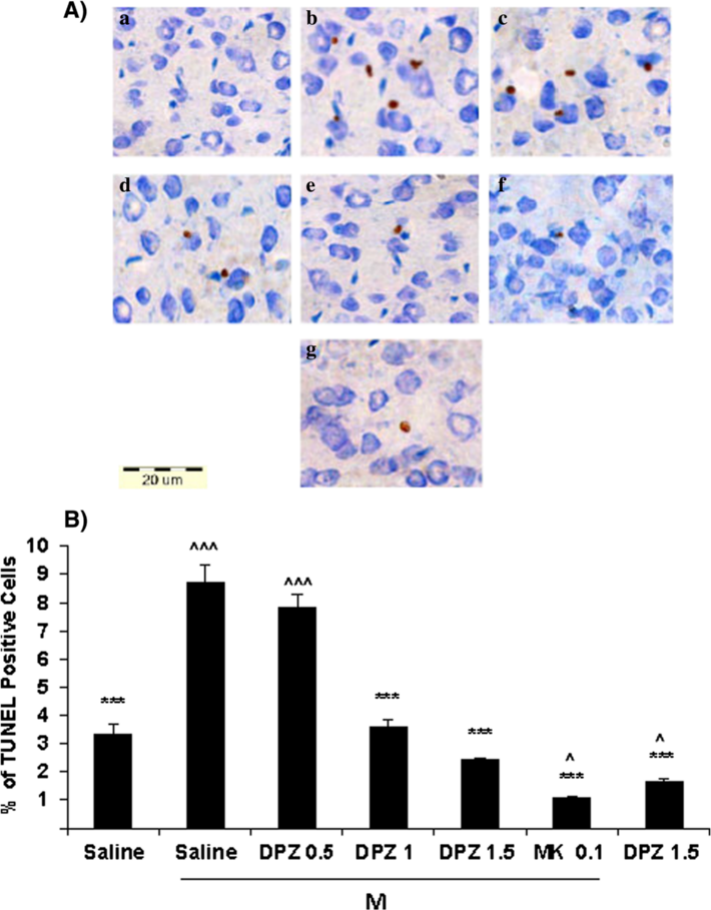
**Effect of daily systemic injections of donepezil (0, 0.5, 1, 1.5 mg/kg, ip) on morphine-induced apoptosis in rat cerebral cortex. A.** Tissue sections from rat’s cerebral cortex were prepared and assayed with an In Situ Cell Death Detection Kit, POD. Slides were counterstained with toluidine blue. Representative photos illustrate the subpopulation of apoptotic cells, which are scattered throughout the tissue section and were intensely stained (brown) by the TUNEL treatment. Slides were analyzed with a light microscope (40× objective). **a)** Normal saline (1 ml/kg/d ip, for 14 days). **b)** Morphine (10 mg/kg/d ip, for 14 days) + donepezil (0 mg/kg, ip for 14 days). **c)** Morphine + Donepezil (0.5 mg/kg ip, for 14 days). **d)** Morphine + Donepezil (1 mg/kg ip, for 14 days). **e)** Morphine + Donepezil (1.5 mg/kg ip, for 14 days). **f)** Donepezil (1.5 mg/kg ip, for 14 days). **g)** Morphine + MK801(0.1 mg/kg ip, for 14 days). **B.** Quantification of apoptotic cells in rat cerebral cortexes. The data represent the mean ± sem number of apoptotic (terminal deoxynucleotidyl transferase-mediated dUTP nick-end labeling [TUNEL] positive) cells in 30 fields, which were counted at a magnification of 40× with a light microscope. A one-way analysis of variance (ANOVA) followed by Tukey’s test was used to analyze the statistical significances. The Scale bar represents a length of 20 μm. A *P* value of <0.05 was considered significant for all analyses. ***P < 0.001 in comparison with the corresponding morphine plus saline (M + S) group. ^^^*P* < 0.001 in comparison with the corresponding saline group. ^*P* < 0.05 in comparison with the corresponding saline group. DPZ = Donepezil, M = Morphine.

**Figure 5:**
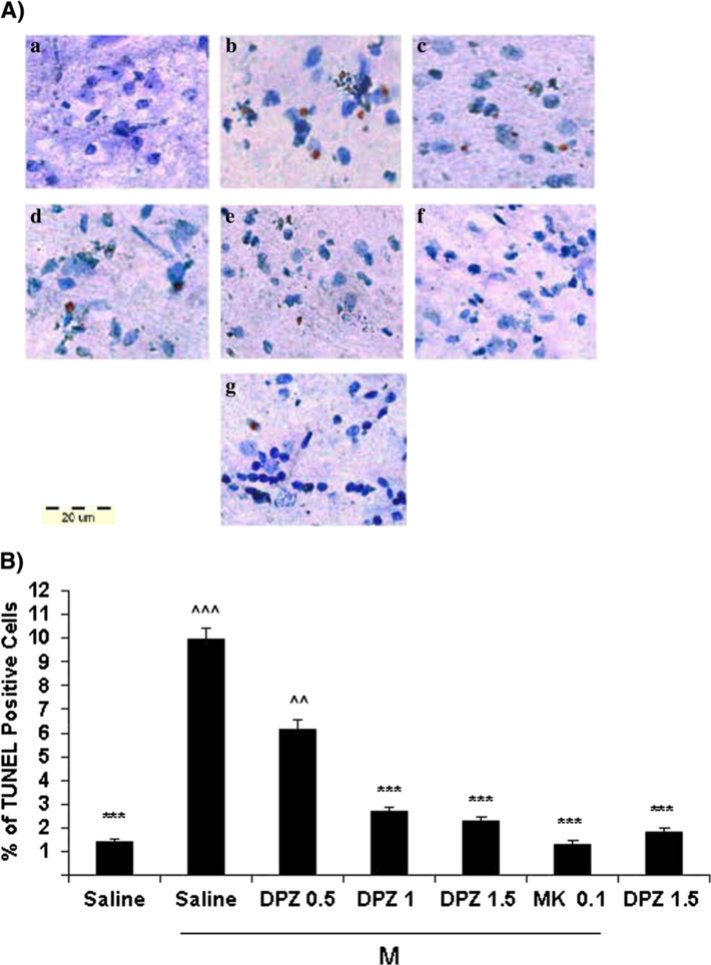
**Effect of daily systemic injections of donepezil (0, 0.5, 1, 1.5 mg/kg, ip) on morphine-induced apoptosis in rat lumbar spinal cord. A.** Tissue sections from rat lumbar spinal cord were prepared and assayed with an In Situ Cell Death Detection Kit, POD. Slides were counterstained with toluidine blue. Representative photos illustrate the subpopulation of apoptotic cells, which are scattered throughout the tissue section and were intensely stained (brown) by the TUNEL treatment. Slides were analyzed with a light microscope (40× objective). **a)** Normal saline (1 mL/kg/d ip, for 14 days). **b)** Morphine (10 mg/kg/d ip, for 14 days) + donepezil (0 mg/kg, ip for 14 days). **c)** Morphine + Donepezil (0.5 mg/kg ip, for 14 days). **d)** Morphine + Donepezil (1 mg/kg ip, for 14 days). **e)** Morphine + Donepezil (1.5 mg/kg ip, for 14 days). **f)** Donepezil (1.5 mg/kg ip, for 14 days). **g)** Morphine + MK801(0.1 mg/kg ip, for 14 days). **B.** Quantification of apoptotic cells in rat lumbar spinal cords. The data represent the mean ± sem number of apoptotic (terminal deoxynucleotidyl transferase-mediated dUTP nick-end labeling [TUNEL] positive) cells in 30 fields, which were counted at a magnification of 40 × with a light microscope. A one-way analysis of variance (ANOVA) followed by Tukey’s test was used to analyze the statistical significances. The Scale bar represents a length of 20 μm. A *P* value of <0.05 was considered significant for all analyses. ***P < 0.001 in comparison with the corresponding control group. ^^^*P* < 0.001 in comparison with the corresponding saline group. ^^*P* < 0.01 in comparison with the corresponding saline group. DPZ = Donepezil, M = Morphine.

The TUNEL method was used to identify apoptotic cells. In the control group (morphine + saline), the number of TUNEL-positive cells significantly (p < 0.001) increased in cerebral cortex (Figure 4) and spinal cord (Figure 5) in comparison with vehicle-treated animals, which indicating that there was an increased basal level of apoptosis in morphine-treated animals. On the other hand the average number of TUNEL-positive cells in the cerebral cortex and lumbar spinal cord was significantly reduced in the donepezil groups (1 and 1.5 mg/kg) compared with those in the control group. Furthermore there were significant differences in the number of TUNEL-positive cells between animals that had received vehicle or donepezil without morphine *versus* those that received morphine (p *<* 0.001). Also the results showed that the TUNEL-positive cells have been attenuated in animals treated with the higher dose of donepezil (1.5 mg/kg) compared to the lower doses. In addition the number of apoptotic cells were clearly decreased in rats received a combination of morphine and MK-801 (0.1 mg/kg, a noncompetitive NMDAR antagonist) in comparison with the control group (Figures 4 and 5).

## Discussion

The results of the present study showed that chronic administration of morphine for 14 days induced tolerance to its analgesic effects, while administration of donepezil (0.5, 1 and 1.5 mg/kg, ip) decreased the development of this tolerance by shifting the first day of established tolerance from the 14th to the 23th, 26th and 31th day respectively. Also the results indicated that there was a significant shift to the left in the dose–response curve as well as a decrease in the antinociceptive 50% effective dose (ED_50_) of morphine for animals who received morphine and donepezil (1.5 mg/kg) compared to the control which means that donepezil prevented the shifting of dose–response curve and ED50 to the right. Moreover, administration of donepezil (1.5 mg/kg) alone had no significant analgesic effect (Additional file 1) which means that donepezil was not simply enhancing morphine analgesia through an additive mechanism.

Over a decade, it has been reported that chronic morphine administration can increase glutamate release in the CNS [15,27]. Importantly, excessive release and accumulation of glutamate, which is associated with an increase in the level of intracellular calcium, plays an important role in CNS injury and neurodegenerative diseases [28].

Several lines of evidence suggest that N-methyl-D-aspartate (NMDA) glutamate receptors (NMDARs) are involved in the plasticity that arises from long-term administration of morphine [15,29-31]. The initial evidence supporting this idea was provided by Trujillo and Akil who reported that the NMDA receptor antagonist, MK-801, inhibited the development of tolerance to the antinociceptive effect of morphine without affecting acute morphine antinociception [29]. After this discovery, numerous studies have demonstrated that a variety of NMDA receptor antagonists have the ability to inhibit the development of opiate tolerance and dependence [11,14,15,29-32]. In this study the effect of donepezil was compared to MK801. MK801 is an NMDA receptor antagonist with well-known neuroprotective effect that prevented tolerance to the analgesic effect of opioids so we used this agent as a gold standard.

On the other hand, in the cerebrocortical nerve terminals, donepezil has been found to decrease in glutamate-induced Ca^2+^ influx in the cerebral cortex and the spinal cord of the rat [21]. It has also been reported that high concentrations of donepezil can attenuate excitatory amino acid receptor activation and decrease the excitability of the postsynaptic cell membrane [33,34].

Previous studies demonstrated that both morphine tolerance and the associated neuronal apoptosis share a common cellular mechanism. In line with these findings, it has been reported that MK-801 blocks both tolerance and apoptosis [2]. Furthermore, activation of NMDARs has been shown to initiate intracellular pathways leading to apoptotic cell death. Glutamate and NMDA can cause intracellular Ca^2+^ influx, activation of Ca^2+^-dependent enzymes such as nitric oxide synthase and production of toxic oxygen radicals leading to cell death [35].

Our results in the present study showed that prolonged exposure to morphine induced apoptotic cell death in the cerebral cortex and lumbar spinal cord. These findings confirmed the results of us and others, indicating that chronic morphine administration significantly increases apoptosis in the rat cerebral cortex and lumbar region of the spinal cord [7-9]. On the other hand, co-administration of donepezil and morphine delayed the onset of morphine-induced tolerance and significantly decreased the average number of TUNEL-positive cells.

Other studies have demonstrated that chronic treatment of rats with morphine (to induce tolerant and dependent states) is associated with a remarkable up-regulation of the pro-apoptotic Fas receptor, as well as intracellular pro-apoptotic elements such as caspase-3, combined with an opposing moderate down-regulation of the anti-apoptotic oncoprotein Bcl-2 [2,6]. Although our findings showed the beneficial effect of donepezil on morphine-induced apoptosis but to clarify the cellular mechanisms and identify the markers involved in apoptosis pathways, further studies are needed.

It is well-known that opioid administration is often accompanied by peripheral and central nervous system anticholinergic side effects, such as dry mouth, constipation, urinary hesitancy, sedation, sleep disruption, and respiratory depression. In a previous study donepezil was reported to be useful in the treatment of daytime sedation, associated with the use of opiate analgesics in cancer patients [36]. From the clinical point of view, donepezil as a cholinergic stimulating drug which has shown to be very well tolerated in patients is a promising agent for attenuating tolerance and sedation as two common and potentially dose-limiting side effects of the opiate analgesics. Therefore it is recommended to study the clinical effectiveness of donepezil along with opioids in cancer patients.

## Conclusion

In conclusion, we found that donepezil as a neuroprotective agent prevented morphine-induced tolerance to the analgesic effects. Also it has been concluded that donepezil could attenuate apoptosis in the cerebral cortex and lumbar spinal cord which might be in association with behavioral effects.

## Competing interests

The authors declare that they have no competing interests.

## Authors’ contributions

MS: contribution in doing the experiments. EI: contribution in study design and manuscript preparation. BN: contribution in data analysis and manuscript preparation. SZ: contribution in study design. AA: contribution in doing the experiments and manuscript preparation. KH: contribution in doing the experiments. FM: contribution in doing the experiments. KH: contribution in study design, data analysis and manuscript preparation. All authors read and approved the final manuscript.

## Supplementary Material

Additional file 1 **Analgesic effects of daily systemic injections of donepezil (0, 0.5, 1, 1.5 mg/kg, ip).** Each bar represents mean of %MPE ± sem. for 8 rats. Independent student T test was used to analyze the differences between saline and donepezil group. P-value less than 0.05 were considered to be significant. DPZ=Donepezil. Click here for file

## References

[B1] Ahlemeyer B Krieglstein J Stimulation of 5-HT1A receptor inhibits apoptosis induced by serum deprivation in cultured neurons from chick embryo Brain Res 1997 777 179 86 9449427 10.1016/s0006-8993(97)01109-8

[B2] Mao J Sung B Ji RR Lim G Neuronal apoptosis associated with morphine tolerance: evidence for an opioidinduced neurotoxic mechanism J Neurosci 2002 22 7650 7661 12196588 10.1523/JNEUROSCI.22-17-07650.2002 PMC6757968

[B3] Sastry PS Rao KS Apoptosis and the nervous system J Neurochem 2000 74 1 20 10617101 10.1046/j.1471-4159.2000.0740001.x

[B4] Dawson G Dawson SA Goswami R Chronic exposure to kappa-opioids enhances the susceptibility of immortalized neurons (F-11kappa 7) to apoptosis-inducing drugs by a mechanism that may involve ceramide J Neurochem 1997 68 2363 2370 9166729 10.1046/j.1471-4159.1997.68062363.x

[B5] Yin D Mufson RA Wang R Shi Y Fas-mediated cell death promoted by opioids Nature 1999 397 218 10.1038/16612 9930695

[B6] Boronat MA Garcia-Fuster MJ Garcia-Sevilla JA Chronic morphine induces up-regulation of the proapoptotic Fas receptor and down-regulation of the antiapoptotic Bcl-2 oncoprotein in rat brain Br J Pharmacol 2001 134 1263 1270 10.1038/sj.bjp.0704364 11704646 PMC1573055

[B7] Hassanzadeh K Habibi-asl B Roshangar L Nemati M Ansarin M Farajnia S Intracerebroventricular administration of riluzole prevents morphine-induced apoptosis in the rat lumbar spinal cord Pharmacol Rep 2010 62 664 673 20885006 10.1016/s1734-1140(10)70323-6

[B8] Hassanzadeh K Habibi-Asl B Farajnia S Roshangar L Minocycline prevents morphine-induced apoptosis in rat cerebral cortex and lumbar spinal cord: a possible mechanism for attenuating morphine tolerance Neurotox Res 2011 19 649 659 10.1007/s12640-010-9212-0 20711699

[B9] Hassanzadeh K Roshangar L Habibi-asl B Farajnia S Izadpanah E Nemati M Arasteh M Mohammadi S Riluzole prevents morphine-induced apoptosis in rat cerebral cortex Pharmacol Rep 2010 62 664 673 21857080 10.1016/s1734-1140(11)70581-3

[B10] Mayer DJ Mao J Holt J Price DD Cellular mechanisms of neuropathic pain, morphine tolerance, and their interactions Proc Natl Acad Sci USA 1999 96 7731 7736 10.1073/pnas.96.14.7731 10393889 PMC33610

[B11] Habibi-Asl B Hassanzadeh K Khezri E Mohammadi S Evaluation the effects of dextromethorphan and midazolam on morphine induced tolerance and dependence in mice Pak J Biol Sci 2008 11 1690 1695 10.3923/pjbs.2008.1690.1695 18819620

[B12] Gracy KN Svingos AL Pickel VM Dual ultrastructural localization of mu-opioid receptors and NMDA-type glutamate receptors in the shell of the rat nucleus accumbens J Neurosci 1997 17 4839 4848 9169542 10.1523/JNEUROSCI.17-12-04839.1997 PMC6573336

[B13] Habibi-Asl B Hassanzadeh K Vafai H Mohammadi S Development of morphine induced tolerance and withdrawal symptoms is attenuated by lamotrigine and magnesium sulfate in mice Pak J Biol Sci 2009 12 798 803 10.3923/pjbs.2009.798.803 19806811

[B14] Habibi-Asl B Hassanzadeh K Moosazadeh S Effects of ketamine and magnesium on morphine induced tolerance and dependence in mice DARU 2005 13 110 115

[B15] Trujillo KA The neurobiology of opiate tolerance, dependence and sensitization: mechanisms of NMDA receptor-dependent synaptic plasticity Neurotox Res 2002 4 373 391 10.1080/10298420290023954 12829426

[B16] Rothman SM Olney JW Glutamate and the pathophysiology of hypoxic-ischemic brain damage Ann Neurol 1986 19 105 111 10.1002/ana.410190202 2421636

[B17] Salinska E Danysz W Lazarewicz JW The role of excitotoxicity in neurodegeneration Folia Neuropathol 2005 43 322 339 16416396

[B18] Mao J Price DD Zhu J Lu J Mayer DJ The inhibition of nitric oxide-activated poly(ADP-ribose) synthetase attenuates transsynaptic alteration of spinal cord dorsal horn neurons and neuropathic pain in the rat Pain 1997 72 355 366 10.1016/S0304-3959(97)00063-8 9313276

[B19] Whiteside GT Munglani R Cell death in the superficial dorsal horn in a model of neuropathic pain J Neurosci Res 2001 64 168 173 10.1002/jnr.1062 11288144

[B20] Habibi-Asl B Hassanzadeh K Charkhpour M Central administration of minocycline and riluzole prevents morphine-induced tolerance in rats Anesth Analg 2009 109 936 942 10.1213/ane.0b013e3181ae5f13 19690270

[B21] Kihara T Shimohama S Sawada H Honda K Nakamizo T Shibasaki H Kume T Akaike A: alpha 7 nicotinic receptor transduces signals to phosphatidylinositol 3-kinase to block A beta-amyloid-induced neurotoxicity J Biol Chem 2001 276 13541 13546 11278378 10.1074/jbc.M008035200

[B22] Takada Y Yonezawa A Kume T Katsuki H Kaneko S Sugimoto H Akaike A Nicotinic acetylcholine receptor-mediated neuroprotection by donepezil against glutamate neurotoxicity in rat cortical neurons J Pharmacol Exp Ther 2003 306 772 777 10.1124/jpet.103.050104 12734391

[B23] Hashimoto M Kazui H Matsumoto K Nakano Y Yasuda M Mori E Does donepezil treatment slow the progression of hippocampal atrophy in patients with Alzheimer’s disease? Am J Psychiatry 2005 162 676 682 10.1176/appi.ajp.162.4.676 15800138

[B24] Asomugha C Linn D Linn C ACh receptors link two signaling pathways to neuroprotection against glutamate-induced excitotoxicity in isolated RGCs J Neurochem 2010 112 214 226 10.1111/j.1471-4159.2009.06447.x 19845831 PMC2809138

[B25] D’ Amour FE Smith DL A method for determining loss of pain sensation J Pharmacol Exp Ther 1941 72 74 79

[B26] McCarthy RJ Kroin JS Tuman KJ Penn RD Ivankovich AD Antinociceptive potentiation and attenuation of tolerance by intrathecal co-infusion of magnesium sulfate and morphine in rats Anesth Analg 1998 86 830 836 9539610 10.1097/00000539-199804000-00028

[B27] Bobula B Hess G Effects of morphine and methadone treatments on glutamatergic transmission in rat frontal cortex Pharmacol Rep 2009 61 1192 1197 20081256 10.1016/s1734-1140(09)70183-5

[B28] Wang SJ Wang KY Wang WC Mechanisms underlying the riluzole inhibition of glutamate release from rat cerebral cortex nerve terminals (synaptosomes) Neuroscience 2004 125 191 201 10.1016/j.neuroscience.2004.01.019 15051158

[B29] Trujillo KA Akil H Inhibition of morphine tolerance and dependence by the NMDA receptor antagonist MK-801 Science 1991 251 85 87 10.1126/science.1824728 1824728

[B30] Trujillo KA Effects of non-competitive N-methyl-d-aspartate receptor antagonists on opiate tolerance and physical dependence Neuropsychopharmacol 1995 13 301 307 10.1016/0893-133X(95)00088-U 8747754

[B31] Mao J NMDA and opioid receptors: their interactions in antinociception, tolerance and neuroplasticity Brain Res Rev 1999 30 289 304 10.1016/S0165-0173(99)00020-X 10567729

[B32] Habibi-Asl B Hassanzadeh K Effects of ketamine and midazolam on morphine induced dependence and tolerance in mice DARU 2004 12 101 105

[B33] Cao YJ Dreixler JC Couey JJ Houamed KM Modulation of recombinant and native neuronal SK channels by the neuroprotective drug riluzole Eur J Pharmacol 2002 449 47 54 10.1016/S0014-2999(02)01987-8 12163105

[B34] Centonze D Calabresi P Pisani A Marinelli S Marfia GA Bernardi G Electrophysiology of the neuroprotective neuroprotective agent riluzole on striatal spiny neurons Neuropharmacol 1998 37 1063 1070 10.1016/S0028-3908(98)00081-1 9833635

[B35] Tamura Y Sato Y Akaike A Shiomi H Mechanisms of cholecystokinin-induced protection of cultured cortical neurons against N-methyl-D-aspartate receptor-mediated glutamate cytotoxicity Brain Res 1992 592 317 325 10.1016/0006-8993(92)91691-7 1360313

[B36] Slatkin NE Rhiner M Treatment of opiate-related sedation: utility of the cholinesterase inhibitors J Support Oncol 2003 1 53 63 15352644

