# Supplementary material for: A new pharmacological role for donepezil: attenuation of morphine-induced tolerance and apoptosis in rat central nervous system
Source: J Biomed Sci. 2014 Jan 23;21(1):6. doi: 10.1186/1423-0127-21-6 (PMC3906771; doi:10.1186/1423-0127-21-6)
Supplement: Additional file 1 — Analgesic effects of daily systemic injections of donepezil (0, 0.5, 1, 1.5 mg/kg, ip). Each bar represents mean of %MPE ± sem. for 8 rats. Independent student T test was used to analyze the differences between saline and donepezil group. P-value less than 0.05 were considered to be significant. DPZ=Donepezil. [file 1423-0127-21-6-S1.doc]

**
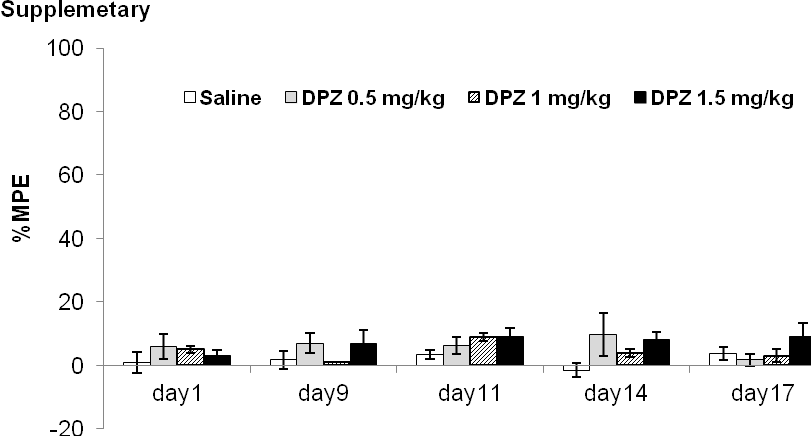
**

Analgesic effects of daily systemic injections of donepezil (0, 0.5, 1, 1.5 mg/kg, ip). Each bar represents mean of %MPE ± sem. for 8 rats. Independent student T test was used to analyze the differences between saline and donepezil group. P-value less than 0.05 were considered to be significant. DPZ=Donepezil.
